# Active Surveillance for Influenza A Virus among Swine, Midwestern United States, 2009–2011

**DOI:** 10.3201/eid1906.121637

**Published:** 2013-06

**Authors:** Cesar A. Corzo, Marie Culhane, Kevin Juleen, Evelyn Stigger-Rosser, Mariette F. Ducatez, Richard J. Webby, James F. Lowe

**Affiliations:** University of Minnesota, Saint Paul, Minnesota, USA (C.A. Corzo, M. Culhane, K. Juleen);; St. Jude Children’s Research Hospital, Memphis, Tennessee, USA (E. Stigger-Rosser, M.F. Ducatez, R.J. Webby);; Carthage Veterinary Services, Carthage, Illinois, USA (J.F. Lowe)

**Keywords:** Influenza, surveillance, monitoring, swine, viruses, midwestern United States

## Abstract

Veterinary diagnostic laboratories identify and characterize influenza A viruses primarily through passive surveillance. However, additional surveillance programs are needed. To meet this need, an active surveillance program was conducted at pig farms throughout the midwestern United States. From June 2009 through December 2011, nasal swab samples were collected monthly from among 540 groups of growing pigs and tested for influenza A virus by real-time reverse transcription PCR. Of 16,170 samples, 746 were positive for influenza A virus; of these, 18.0% were subtype H1N1, 16.0% H1N2, 7.6% H3N2, and 14.5% (H1N1)pdm09. An influenza (H3N2) and (H1N1)pdm09 virus were identified simultaneously in 8 groups. This active influenza A virus surveillance program provided quality data and increased the understanding of the current situation of circulating viruses in the midwestern US pig population.

Influenza A virus has become a major pathogen, causing epidemics of respiratory disease in humans, which not only result in increased deaths but also raise public health organization alarms regarding the need for further understanding and control of this virus (*1*). Additionally, the ability of the virus to cross species barriers has raised more concern over the probability of reassortment and generation of highly transmissible viruses that might pose a threat to humans (*2*). Despite evidence of reassortment in other species, swine have been most often labeled as the “mixing vessel” because avian- and mammalian-type receptors for influenza A virus have been found in pig tracheas, making swine a potential source of new viruses through reassortment (*3**,**4*). Because these viruses can infect humans, influenza A virus in swine should be monitored for public health reasons (*5*).

In the United States, influenza A virus has been present in swine for almost a century (*6*). Within the US pig population, 3 major subtypes of influenza A virus (H1N1, H1N2, H3N2) circulate, causing widespread respiratory disease characterized by dry coughing, sneezing, fever, anorexia, rhinorrhea, and lethargy (*7*). Swine influenza viruses have been monitored through seroprevalence studies. Such studies from the 1970s through the 1990s revealed that influenza virus subtypes H1N1 and H3N2 circulated in the US pig population (*8**–**11*).

At the turn of the 21st century, 2 new viruses were detected in the swine population. These viruses were the result of either double or triple reassortment between human, avian, and swine viruses (*11**–**13*). Since 1998, circulating influenza viruses in pigs have been able to change because of mutations and the propensity for influenza A virus of swine with the triple reassortant genotype to frequently reassort and generate new genotypes, therefore increasing the diversity of influenza A virus in swine (*14*). Virologic and seroprevalence studies have provided valuable information about influenza A virus in swine, but the epidemiology of influenza A virus in swine is not fully understood.

Newly emerged pathogens can be detected through passive or active surveillance. Passive surveillance is driven by laboratory submission of samples after outbreaks of respiratory disease, whereas active surveillance is based on purposely collecting and screening field samples regardless of clinical status. In Asia, active surveillance for influenza conducted through collection of nasal swabs at slaughter plants has reportedly detected uncommon influenza viruses (i.e., subtypes H3N1, H7N2, H9N2) in the local pig population (*15**–**17**)*. In the United States, similar studies, following the same sample collection method, during the early and late 1990s have been reported (*10**,**18*). However, a surveillance program that will identify and report newly emerged viruses in a timely manner is still needed (*14*).

Overall, studies have elucidated epidemiologic features of the virus in swine, such as the constant circulation of influenza A virus in the swine population and sporadic infections with rare subtypes. However, absence of a proactive approach leaves a gap that needs to be filled (*19*). Therefore, the objectives of this study were to 1) conduct an active surveillance program to better characterize the presence of influenza viruses in the swine population and 2) make live viruses available for genetic characterization, potential vaccine, and diagnostic use. Procedures and protocols used in this study were approved by the University of Minnesota Institutional Animal Care and Use Committee

## Methods

### Farm Selection

 Veterinarians who agreed to participate in the study were asked to enroll growing-pig farms (i.e., farms that house pigs 3–30 weeks of age) in the midwestern United States that were representative of modern swine production systems and that were owned by producers interested in participating in the study. Producers were allowed to withdraw from the study at any time.

### Sample Collection

From June 2009 through December 2011, participating farms were visited every month for 12–24 consecutive months. At each visit, the investigator would meet with the farm manager/owner to decide which pigs were to be sampled. If pigs were all in 1 age group, samples were collected from that group; if pigs were in >1 age group, samples would be collected from the age group closest to 10 weeks, the group most likely to yield the most influenza A virus–positive pigs, per previous reports (*20*).

A total of 30 nasal swab samples were collected at each visit, enabling us to be 95% confident of detecting at least 1 positive sample when influenza prevalence was at least 10%. Clinically healthy pigs were restrained by a snare, and a nasal swab (Starswab II, Starplex Scientific Inc., Etobicoke, Ontario, Canada) was inserted 2–3 inches into the back of each nostril while being rotated. Nasal swabs were labeled with a specific code containing the farm identification number, month, 2-letter state abbreviation, and nasal swab sample number.

During the visit, the age of the pigs and respiratory clinical signs (absence or presence of sneezing, coughing, and nasal secretion) among the group members were recorded. Nasal swabs and submission sheets were placed into a Styrofoam container with ice packs and shipped overnight to the laboratory for testing.

### Sample Testing

All nasal swab samples were tested at the virology department laboratory of St. Jude Children’s Research Hospital (Memphis, TN, USA). Nasal swab samples were initially screened for influenza A virus by real-time reverse transcription (RRT-PCR) selective for the matrix gene. Samples that were positive by RRT-PCR underwent further diagnostics for determination of subtype, including the influenza A(H1N1)pdm09 virus and swine H1 and H3 viruses (*21**–**23*).

### Statistical Analyses

A farm was considered positive for a given month if any of the 30 individually collected swab samples tested positive. Farm-level data, such as farm size, were analyzed by repeated measures logistic regression, and differences between farms were accounted for by including farm as a random effect and allowing for an autoregressive effect by month within the farm. Model building was performed by first screening independent variables through univariate analysis. Variables with a p value <0.25 were retained for the multivariable model. All selected variables were forced in the model including 2-way interactions and were sequentially removed if p value was >0.05.

We built 2 models. The first model assessed the relationships between farm status (positive vs. negative) for influenza virus and age, year, clinical signs, and season. The second model assessed the relationship between respiratory clinical signs (presence vs. absence) and subtype and season. Season was included in the models by categorizing the 4 seasons as follows: winter (January–March), spring (April–June), summer (July–September), and fall (October–December). Statistical procedures were performed in SAS 9.2 (SAS Institute Inc., Cary, NC, USA).

## Results

Farms were enrolled in the program as agreement to participate (by veterinarians and producers) was obtained; thus, the program started in June 2009 and ended in December 2011. The 33 producers who agreed to participate were located throughout the midwestern United States: 17 farms in Iowa, 4 in Illinois, 8 in Indiana, and 4 in Minnesota. Each of the 33 farms housed 1,000–13,000 pigs. One group of 7 farms in Iowa withdrew from the program in August 2009 after the influenza A(H1N1)pdm09 virus–associated crisis in the swine industry.

### Sample Test Results

**Figure 1:**
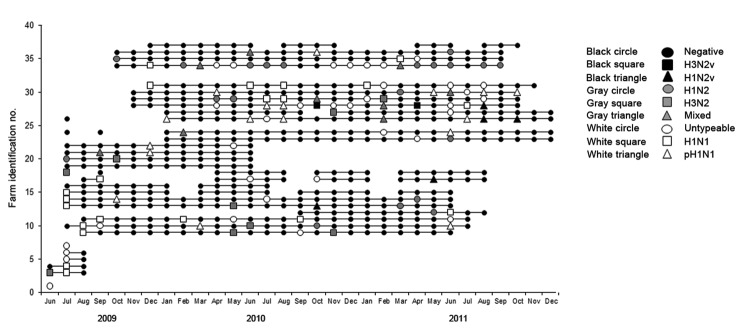
Swine influenza virus group status for 32 pig farms participating in an active surveillance project, midwestern United States, June 2009–December 2011. Each horizontal line represents a farm, each dot represents a sampling event, and colors indicate virus status of the group.

A total of 16,170 nasal swab samples from 540 groups of growing pigs from the remaining 32 farms were collected. From the total number of samples collected, 746 (4.6%) were positive for influenza A virus by RRT-PCR, and 178 viruses were isolated from these samples. At least 1 positive sample was detected in 117 (21.7%) groups of pigs; thus, these groups were classified as positive. Of the 32 farms, 29 (90.6%) had at least 1 positive group throughout the study. Of the 117 groups with RRT-PCR–positive results for influenza A virus, complete or partial subtype details were obtained for 99 (84.6%) pig groups (Figure 1). Influenza A virus infection with just 1 subtype (H1N1, H1N2, H3N2, or H1N1pdm09) was detected in 21, 19, 9, and 17 groups, respectively. Dual infections were detected in 10 groups, of which 8 concurrently harbored influenza virus subtypes H1N2 and H1N1pdm09, 1 harbored subtype H1N1 and an H3N-untypeable virus, and the remaining group harbored subtype H1N1 and an H1N-untypeable virus. Partial subtyping information was obtained for 16 pig groups, from which 11, 4 and 1 were infected with an H1N-untypeable, H3N-untypeable, and an H1N-untypeable with pandemic matrix gene virus subtype, respectively. In 18 groups, a subtype could not be defined through either RRT-PCR or sequencing. At most farms in our study, groups of pigs were identified as having multiple and different influenza A viruses detected throughout the surveillance period (Figure 1). Viruses were isolated from 178 swab samples that originated from 62 pig groups.

**Figure 2:**
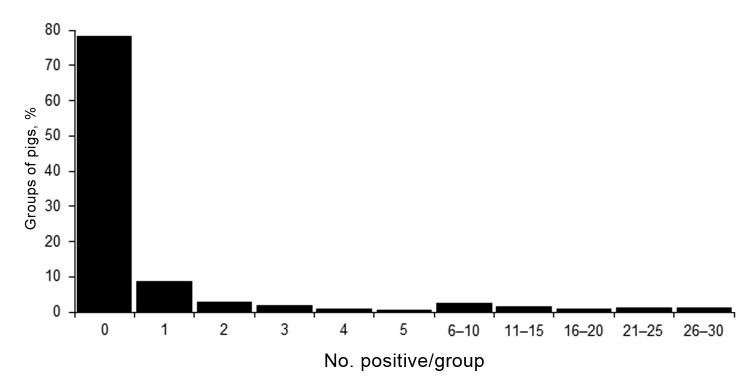
Frequency distribution of number of nasal swab samples positive for influenza virus by real-time reverse transcription PCR, per group (total 540 groups of pigs), midwestern United States, June 2009–December 2011.

Of the positive groups, the mean and median numbers of samples positive for influenza A virus by RRT-PCR were 6.4 and 2, respectively. The numbers of positive samples ranged from 1 to 30; most groups (n = 48) had 1 positive sample (Figure 2). There were 15, 10, 4, and 2 groups that had 2, 3, 4, and 5 positive samples, respectively. A total of 13 groups had >20 positive samples, 2 had 29 positive samples, and 1 had 30 positive samples. Although most samples collected from these 13 groups were positive for influenza A virus by RRT-PCR, pigs in only 7 of these groups exhibited clinical signs of influenza-like illness.

**Figure 3:**
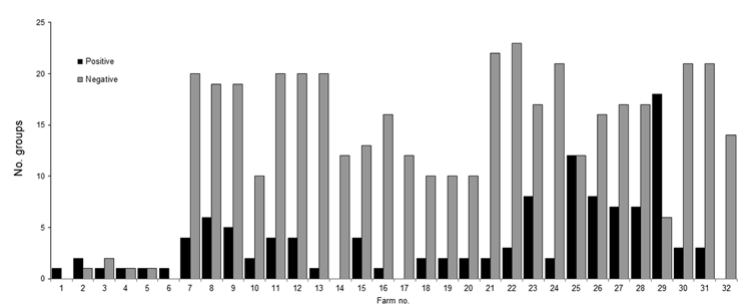
Number of influenza A virus–positive and of influenza A virus–negative groups, by farm, midwestern United States, June 2009–December 2011.

The number of positive groups per farm ranged from 1 to 18. On average, 31% of the groups tested by farm throughout the program were classified as positive (Figure 3). Farms with no influenza A virus–positive results were monitored for ≈1 year but lacked consistency in the testing frequency and time intervals between tests.

**Figure 4:**
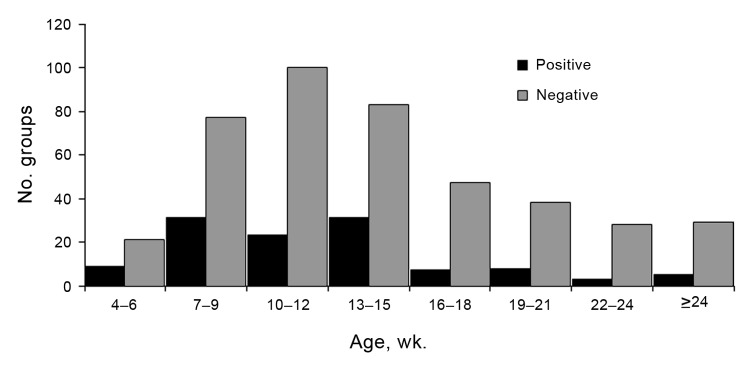
Age distribution of groups of pigs that were positive or negative for influenza A, determined by real-time reverse transcription PCR, midwestern United States, June 2009–December 2011.

The average age of the pigs in the 540 groups was 13.7 ± 5.7 weeks. Influenza virus was detected in pigs as young as 4 weeks and as old as 30–32 weeks of age. Mean age in positive groups was 12.4 ± 5.2 weeks and in negative groups was 13.9 ± 5.8 SD (Figure 4). However, age was not a statistically significant predictor of influenza A virus test status.

### Clinical Signs

Respiratory clinical signs were observed in pigs in 187 (34.6%) of 540 groups. From these 187 groups that reportedly exhibited clinical signs, 43 (22.9%) were positive for influenza A virus. From the 353 groups that exhibited no clinical signs, 74 (20.9%) were positive for influenza virus (Table 1). Even when within-group prevalence of influenza A virus was high, such as in the 13 groups in which >20 samples were positive for influenza A virus, clinical signs of respiratory disease were low. Indeed, clinical signs of influenza-like illness were noted in only 7 of those 13 groups.

**Table 1:** Influenza virus status and respiratory clinical signs among growing pigs, midwestern United States, June 2009–December 2011

Clinical signs	Influenza virus status, no. groups		Total, no. groups
	Positive	Negative	
Present	43	144	187
Absent	74	279	353
Total	117	423	540

### Season

Throughout the study, the numbers (proportions) of groups sampled in each season were similar. In winter, 124 (23%) were sampled; in spring, 137 (25%); in summer, 150 (28%); and in fall, 129 (24%) (Table 2).

**Table 2:** Influenza virus status among growing pigs, by season, midwestern United States, June 2009–December 2011

Season	Influenza virus status, no. groups		Total, no. groups
	Positive	Negative	
Winter	19	105	124
Spring	37	100	137
Summer	40	110	150
Fall	21	108	129
Total	117	423	540

### Logistic Regression

In the first model, univariate analysis of all variables (age, season, and year) except clinical signs yielded p<0.25. The multivariable model retained 2 variables: season and age. Odds of positive results for influenza A virus were 2 (95% CI 1.1–3.8) times and 1.9 (95% CI 1.0–3.5) times higher for groups of pigs tested during the spring and summer, respectively, than for groups tested during the fall. There was no association between the winter season and influenza virus detection. Age was not significantly (p = 0.09) associated with influenza A virus group status; however, the variable was left in the model because of confounding (Table 3).

**Table 3:** Relationship between group influenza status with age and season in growing pigs tested for influenza virus, midwestern United States, June 2009–December 2011*

Variable	Estimate	Odds ratio (95% CI)	p value
Intercept	–1.330	NA	<0.01
Season			
Fall (referent)	NA	NA	NA
Spring	0.741	2.0 (1.1– 3.8)	0.01
Summer	0.657	1.9 (1.0–3.5)	0.03
Winter	–0.072	0.9 (0.4–1.8)	0.83
Age	–0.041	0.9 (0.9–1.0)	0.09

*Repeated measures logistic regression multivariable analysis. Generalized χ^2^ divided by degrees of freedom = 0.92. NA, not applicable.

In the second model, complete subtype information was available for 81 monthly test results. Because neither subtype nor season was significantly associated with presence of clinical signs at the univariate level (Table 4), no attempts were made to build a multivariate model.

**Table 4:** Relationships between clinical signs with subtype and season among pigs tested for influenza virus, midwestern United States, June 2009–December 2011*

Variable	Estimate	Odds ratio (95% CI)	p value
Season†			
Intercept	*–*0.234	NA	0.65
Fall (referent)	NA	NA	NA
Spring	–0.411	0.6 (0.1–2.5)	0.53
Summer	–0.410	0.6 (0.1–2.4)	0.53
Winter	0.361	1.4 (0.3–6.7)	0.63
Influenza virus subtype‡			
Intercept	1.7	NA	0.95
H1N1 (referent)	NA	NA	NA
H1N2	–1.381	0.5 (0.1–2.0)	0.96
H3N2	–1.476	0.5 (0.09–2.5)	0.95
H1N2v	–2.105	0.2 (0.03–2.0)	0.93
H3N2v	9.541	NA	0.95
H1N1pdm09	–1.582	0.4 (0.1–1.7)	0.95
Mixed infection	–2.210	0.2 (0.04–1.3)	0.93

*Logistic regression univariate analysis; NA, not applicable.  †Generalized χ^2^ divided by degrees of freedom = 0.96. ‡Likelihood ratio χ^2^ (6) = 6.01 df, p = 0.42.

## Discussion

Active surveillance at 32 farms demonstrated that influenza A virus is commonly present in the nasal secretions of pigs; 29 (90.6%) farms had at least 1 positive group throughout the study. Despite this high group or population prevalence, detection of swine influenza A virus in individual samples was low and thereby compatible with previously published findings (*10**,**15**–**18*) in which swine influenza A virus detection rates through either virus isolation or RRT-PCR on individual nasal swab samples was ≤5%, presenting a challenge for surveillance programs. However, new sample collection techniques in swine, such as the collection of pen-based oral fluids (e.g., saliva) for antibody and antigen detection are becoming more commonly used because of their practicality and lower testing costs (*24*); other studies have shown an increased probability of detection, making oral fluid sampling a suitable and essential tool for population surveillance on pig farms (*25**,**26*). Asymptomatic carriers of influenza A virus can be detected more efficiently through oral fluid sampling of clinically healthy populations than through nasal swab sampling.

Influenza A virus persistence in pig populations is not fully understood. In our study, 41% of the positive groups had only 1 positive nasal swab sample. One possible explanation could be that these groups of pigs were sampled either at the beginning or end of an infection. Another possible explanation could be that underlying passive or active immunity enabled transmission to occur at a rather low rate and that transmission remained continuous in and thus perpetuated the infection within the population. However, our study was not designed to measure transmission. In addition, our study did not obtain information regarding the entrance into or exit from the farm by groups of pigs. Therefore, it is possible that the same group of pigs was sampled on consecutive months.

In our study, the absence of respiratory clinical signs in groups of pigs harboring influenza A virus is notable. This lack of signs could be the result of events such as the presence of antibodies conferred by colostrum ingestion (e.g., maternal immunity), vaccination, or previous exposure; other studies have suggested that low levels of exposure to virus might preclude clinical signs in pigs (*27**–**29*). Subclinical infections in pigs have public health implications because humans can become infected after coming in contact with apparently healthy pigs that are shedding enough infectious viral particles (*5**,**30**–**32*). Subclinical infections are perhaps one of the most common routes for influenza A virus entry into a pig farm because replacement breeding stock or recently weaned animals are constantly moved within and between states and countries. In fact, there is evidence that movement of pigs might have been the cause of dissemination of certain virus lineages within the United States, and subclinical infections might have played a role (*33*).

Pathogenicity of swine influenza A virus can vary among strains within the same subtype (*34*). From a clinical signs standpoint, experimental infection with influenza A virus has resulted in great variability (*29*). When swine influenza A virus is part of a co-infection (i.e., with other viruses and/or bacteria), clinical signs are evident (*35**,**36*); such co-infections might reflect the health status situation of the groups of pigs used in this study because the likelihood of co-infections in the field was high. However, in our study, the lack of association between virus subtype and clinical signs is difficult to explain. More studies are needed to better understand the role that virus subtypes play on the presence of respiratory signs.

Our study demonstrated that influenza A virus is present in growing pigs throughout the year and that groups of pigs are more likely to have positive test results during the spring and summer than in the fall. This finding is contrary to what has been suggested (*7*). The previously suggested seasonal trend could have been based on presence of clinical signs that appear during a time of the year when other factors are present (e.g., cold weather, bad air quality inside barns, co-infections) (*7**,**37*), which led veterinarians to submit samples to diagnostic laboratories for the detection of influenza A virus. However, as mentioned earlier, subclinical infections might have occurred during warm months, thereby leading to misinterpretation of the information available at that time. Another possible explanation for finding more influenza A virus–positive groups in the spring and summer is the increase of pigs born to primiparous females. This increase in potentially more susceptible growing pigs is a result of increased breeding of gilts (female pigs that have not had their first litter) during late summer, when swine producers often increase the number of gilts bred. These primiparous females often have a lower level of antibody protection to offer to their first litter of piglets. These relatively immunologically naive piglets would often be the group studied in the spring and summer seasons (*38*), providing a source of susceptible individuals in which viruses circulate during these seasons. 

A limitation of our study might be the locations of the farms, which were all in the midwestern United States. Weather conditions and seasons in the midwestern United States might not accurately reflect conditions in other swine-producing areas of the United States, namely, the southeastern and south-central regions. For logistical reasons, neither of these regions was included in this study.

Surveillance will continue to be a useful tool for infectious disease epidemiology because the data it provides will aid in the understanding of the determinants of infection, enabling scientists and practitioners to work toward generation of disease prevention and control strategies (*39*). Surveillance studies contribute to science by generating data about zoonotic and emerging pathogens (*40*). Such contributions are true for influenza A virus in swine because emerging influenza viruses have been identified through surveillance programs (*15**,**16*). In summary, our study has led to the following 3 conclusions: 1) different influenza viruses circulate simultaneously within pig populations; 2) influenza is present in pigs of different ages at a rather low prevalence throughout the year; and 3) subclinical infections are frequent among groups of pigs. More studies are needed to add to understanding of influenza A virus.
